# The Spatial Distribution of the Exocyst and Actin Cortical Patches Is Sufficient To Organize Hyphal Tip Growth

**DOI:** 10.1128/EC.00085-13

**Published:** 2013-07

**Authors:** David Caballero-Lima, Iliyana N. Kaneva, Simon P. Watton, Peter E. Sudbery, C. Jeremy Craven

**Affiliations:** Department of Molecular Biology and Biotechnology, University of Sheffield, Sheffield, United Kingdom

## Abstract

In the hyphal tip of Candida albicans we have made detailed quantitative measurements of (i) exocyst components, (ii) Rho1, the regulatory subunit of (1,3)-β-glucan synthase, (iii) Rom2, the specialized guanine-nucleotide exchange factor (GEF) of Rho1, and (iv) actin cortical patches, the sites of endocytosis. We use the resulting data to construct and test a quantitative 3-dimensional model of fungal hyphal growth based on the proposition that vesicles fuse with the hyphal tip at a rate determined by the local density of exocyst components. Enzymes such as (1,3)-β-glucan synthase thus embedded in the plasma membrane continue to synthesize the cell wall until they are removed by endocytosis. The model successfully predicts the shape and dimensions of the hyphae, provided that endocytosis acts to remove cell wall-synthesizing enzymes at the subapical bands of actin patches. Moreover, a key prediction of the model is that the distribution of the synthase is substantially broader than the area occupied by the exocyst. This prediction is borne out by our quantitative measurements. Thus, although the model highlights detailed issues that require further investigation, in general terms the pattern of tip growth of fungal hyphae can be satisfactorily explained by a simple but quantitative model rooted within the known molecular processes of polarized growth. Moreover, the methodology can be readily adapted to model other forms of polarized growth, such as that which occurs in plant pollen tubes.

## INTRODUCTION

The capacity of the human fungal pathogen Candida albicans to grow in a true hyphal mode is a key virulence attribute allowing invasion of mucosal surfaces to establish both superficial infections in otherwise healthy individuals and life-threatening disseminated infections in vulnerable, immunocompromised patients. C. albicans hyphae grow in a highly polarized fashion from their tips in the same way as other fungal hyphae (1). Recently, there have been considerable advances in our understanding of the mechanism of polarized growth of C. albicans hyphae (reviewed in reference 2), providing an opportunity to use C. albicans as a model to facilitate understanding of the general process of polarized growth in fungal hyphae.

There have typically been two approaches to trying to understand the mechanism of such extreme polarized growth in fungal hyphae (which shares some common features with the process of bud formation in yeasts). In the “bottom-up” approaches, a wealth of information has been accumulated at the detailed molecular level; in the “top-down” approaches, investigators have tended to consider growth in terms of overall physical properties, without knowledge of the detailed molecular cellular biological processes. As noted by Harold (3) and later by Slaughter and Li (4), there has been relatively little progress in bridging the gap between these two levels of description. One very successful model, the vesicle supply center (VSC) model, has gone some way toward combining detailed concepts of cell biology and 3-dimensional (3D) physical calculations (5–7). However, this model predates much of the present knowledge of the details of molecular processes that has emerged from studies of model fungi such as Saccharomyces cerevisiae, Neurospora crassa, or Schizosaccharomyces pombe.

In this paper, we present steps toward reconciling these two approaches by a relatively simple mathematical treatment of the process of hyphal growth that incorporates three well-established molecular cell biological processes: the fundamental role of the spatial pattern of cell wall synthesis in determining hyphal form, the role of the exocyst in determining where the cell wall synthesis enzymes are delivered to the plasma membrane, and the role of endocytic patches in ensuring that these enzymes stay only in a discrete zone of the plasma membrane. We apply the model to the growth of hyphae in the human fungal pathogen C. albicans.

Dissection of the secretory pathway in S. cerevisiae established that polarized growth depends on the delivery of secretory vesicles to the sites of polarized growth (8). The fusion of a vesicle with the plasma membrane achieves three things. First, the membrane of the vesicle is incorporated into the plasma membrane, thus allowing expansion of the plasma membrane. Second, the contents of the vesicles, such as glycosylphosphatidylinositol (GPI)-linked mannoproteins, are released into the extracellular space (9). Third (and crucially), enzymes such as (1,3)-β-glucan synthase (GS), which catalyzes the biosynthesis of (1,3)-β-glucan from UDP-glucose monomers, are embedded in the plasma membrane and are thought to extrude (1,3)-β-glucan polymers into the extracellular space (10, 11). (1,3)-β-Glucan is the major load-bearing polymer of the cell wall (along with chitin) and forms a scaffold for the attachment of (1,6)-β-glucan and GPI-anchored mannoproteins (10, 11). Once extruded into the extracellular space, (1,3)-β-glucan is cross-linked to itself, to chitin, and to (1,6)-β-glucan, forming a complex 3-dimensional mesh. It is often assumed that this process of cross-linking is the reason why the cell wall becomes less deformable in subapical regions as it ages.

It must be emphasized that while tip extension in hyphae requires both the expansion of the plasma membrane and of the cell wall, it is the pattern of cell wall deposition that primarily controls the shape of the hyphal tip. The components of lipid membranes can flow, and thus, the lipid membrane can readily undergo large-scale deformations under the influence of forces, whereas, in contrast, the branched and cross-linked nature of the polysaccharide cell wall means that it is much less readily deformable and that it determines the shape of the cell (10). It is also very important to realize that vesicles, in the main, deliver the wall-building enzymes and not the wall material itself: to use a strong analogy, they deliver the bricklayers and not the bricks. In the context of glucan synthase, the bricks are UDP-glucose monomers. UDP-glucose is formed from glucose-1-phosphate and UTP by the action of UTP-glucose-1-phosphate uridylyltransferase (EC 2.7.7.9), encoded by *UGP1* (12). In S. cerevisiae, this enzyme is cytoplasmic (13), so presumably glucan synthase takes up UDP glucose from the cytoplasm and extrudes the glucan polymer into the extracellular space. Interestingly, a reduction in Ugp1 activity to 10% of normal, resulting in a 50% drop in UDP-glucose levels, had no effect on the growth rate, suggesting that the supply of bricks is not rate-limiting (12). This paper focuses on the consequences of the vesicles delivering new cell wall synthesizing capacity.

Vesicles dock with the exocyst before fusion with the plasma membrane. The exocyst is a multiprotein complex composed of eight evolutionarily conserved proteins: Sec3, Sec5, Sec6, Sec8, Sec10, Sec15, Exo70, and Exo84 (14–17). In S. cerevisiae, temperature-sensitive mutations in exocyst components result in an accumulation of post-Golgi vesicles that are unable to fuse with the plasma membrane (8). Deletion of C. albicans SEC3 is not lethal but leads to an accumulation of vesicles in yeast buds, and upon hyphal induction the hyphal tip reverts to nonpolarized growth after the first septum forms, showing that an intact exocyst complex is required for the polarized growth of hyphae (18). It is thought that in S. cerevisiae, Sec3 and part of the Exo70 pool are located independently of the secretory pathway at sites of polarized growth, while the remaining components are carried on vesicles (14, 19). As vesicles arrive at sites of polarized growth, they are tethered to the plasma membrane by the formation of the complete eight-unit complex as the vesicle-associated exocyst components interact with Exo70 and Sec3 located on the plasma membrane. Thus, it follows that the spatial distribution and rate of vesicle fusion will be determined by the location and density of Exo70 and Sec3. We have shown that in C. albicans, all exocyst components locate to a surface crescent, while vesicle-associated proteins, such as Sec4, Sec2, and Mlc1, localize to a subapical spot (20). Moreover, the vesicle-associated proteins were much more dynamic in FRAP (fluorescence recovery after photobleaching) experiments than the exocyst components. Finally we showed that Sec4 disperses very rapidly upon disruption of the actin cytoskeleton, while the exocyst components are, again, much more stable. Based on these observations, we suggested that in C. albicans it is likely that not all vesicles carry their own complement of exocyst subunits. Rather, while exocyst components might originally be transported to the plasma membrane on vesicles, once delivered, they remain in place to tether other vesicles that do not carry exocyst components.

Enzymes, such as (1,3)-β-glucan synthase, that synthesize the main components of the cell wall are delivered to the plasma membrane by vesicles and continue to synthesize the cell wall until they are removed or negatively regulated. Thus, it is necessary to consider how cell wall expansion will lead to the movement of the enzymes away from the region of vesicle fusion and how the molecules are ultimately removed from the membrane. The likely mechanism for this removal is endocytosis, which is thought to occur at the actin cortical patches (21). In C. albicans, as in other fungi that form hyphae, the patches form a subapical collar (22–25). Endocytosis is required for hyphal growth, as evidenced by the fact that a variety of different mutations that interfere with endocytosis either abolish hyphal growth completely or result in abnormal hyphae with swollen tips (26–31). Similarly, treatment of hyphae with the actin-disrupting drug cytochalasin A results in hyphal tip swelling (23, 32).

It is assumed that in S. cerevisiae, the location of Sec3 and Exo70 is determined by the distribution of active GTP-bound Cdc42, and there has been much interest recently in how this Cdc42 distribution is established in the absence of external cues such as the bud site selection pathway (33–36). In this paper, we are not concerned with how the sites of polarized growth are established; instead, we take as our starting point the empirical fact that Exo70 and Sec3 localize to a crescent at the hyphal tip, where we can measure their intensity distribution. We present a model of hyphal tip growth in which secretory vesicles fuse to the plasma membrane at a rate proportional to the local concentration of Exo70 and Sec3, which, based on concurrently available evidence in both S. cerevisiae and C. albicans, determines the distribution of vesicle docking. Because Exo70 and Sec3 localize to the plasma membrane independently of vesicles in S. cerevisiae, we monitored these exocyst components as reporters of where vesicles dock in C. albicans. However, based on our previous work, we suspect that the other exocyst components may also be located at the cell surface rather than being carried on incoming vesicles (20). In our model, the cell wall-synthesizing enzymes, such as glucan synthase, that are consequently inserted continue to generate new cell wall material until they are internalized by endocytosis at the subapical bands of actin cortical patches.

To make the treatment quantitative, we divide the hyphal surface into a set of concentric annular regions. Within each annular region, we calculate the amount of synthase that will be deposited in the plasma membrane during a small interval of time, as secretory vesicles fuse at a rate determined by the local exocyst concentration. We also calculate the enlargement of the cell wall area that will occur during the same interval of time due to the activity of synthase molecules. Based on simple geometry, we calculate the resulting change in the shape of the hypha, assuming that it remains inflated by turgor pressure. We iterate this process forward in time and record the development of the size of each annulus and of the hypha as a whole.

By focusing on the development of annular regions as they develop at the tip, enlarge, and eventually become fixed in size in the parallel walls of the hyphal tube, we make it possible to maintain a close intuitive link between the mathematics of the calculations and the underlying physical and biological phenomena. The direct computational (i.e., numerical) method allows great flexibility in the choice of the rules that determine the rate of change of the cell surface area and prevents the mathematics from being highly specialized. Simplifying assumptions can remain obscured in more complex treatments, and a clear understanding of the existence and nature of these assumptions can be key to deciding where future experimentation should be directed. The equations can be used to calculate the development of the hyphal form (both in time and in 3D space) in a very straightforward manner. We have used the Python programming language (www.python.org), which is freely available across many different computing platforms and is highly regarded for its accessibility to nonspecialists. The simple nature of the computing method also facilitates the addition in the future of extra details, or modifications, as pertinent experimental data become available. Moreover, the methodology can be readily adapted to model other forms of polarized growth, such as those occurring in plant pollen tubes, animal cells, and axons. However, care must be taken to recognize that there may be fundamental differences between these systems. In fungal growth, we have emphasized here that vesicles deliver the capacity to synthesize the cell wall, whereas in pollen tubes, the vesicles deliver the cell wall itself in the form of pectin. We have made careful measurements in living C. albicans cells of the density of fluorescence from exocyst proteins fused to yellow fluorescent protein (YFP). We show that when these measurements are incorporated into the model, the predicted forms of the hyphae are in good accord with experimental observations only if a mechanism exists to remove synthase from the membrane (or downregulate its activity) at a position that is in good accord with the locations of the experimentally observed collars of actin patches. We further show that the distribution of active GS, as reported by green fluorescent protein (GFP) fusions with Rho1, the positive regulatory subunit of GS, and with its guanine-nucleotide exchange factor (GEF) Rom2, is much more extensive than the distribution of exocyst components, to a degree that is well predicted by the model.

## MATERIALS AND METHODS

### Strains.

All strains were derived from BWP17. C-terminal fusions were generated as described previously using pFA-XFP plasmids carrying the appropriate *URA3*, *ARG4*, or *HIS1* genes (37). An N-terminal GFP fusion of Rho1 was generated as described previously using the pFA-HIS1 pMAL-GFP plasmids (38). Full genotypes of the strains are provided in Table 1, and the oligonucleotides used are listed in Table S1 in the supplemental material.

**Table 1:** Strains used in this study

Strain	Genotype	Source or reference
BWP17	*URA3*::λ*imm434/URA3*::λ*imm434 his1*::*hisG/his1*::*hisG arg4*::*hisG/arg4*::*hisG*	54
Exo70-YFP	BWP17 *EXO70/EXO70-YFP*::*URA3*	20
Sec3-YFP	BWP17 *SEC3/SEC3-YFP*::*URA3*	20
Exo84-YFP	BWP17 *EXO84/EXO84-YFP*::*URA3*	20
Sec6-YFP	BWP17 *SEC6/SEC6-YFP*::*URA3*	20
Sec8-YFP	BWP17 *SEC8/SEC8-YFP*::*URA3*	20
Abp1-YFP	BWP17 *ABP1/ABP1-YFP*::*URA3*	23
Rom2-GFP	BWP17 *ROM2/ROM2-GFP*::*URA3*	This study
Rom2-GFP/GFP	BWP17 *ROM2-GFP*::*URA3*/*ROM2-GFP*::*ARG4*	This study
GFP-Rho1	BWP17 *RHO1*/*GFP-RHO1*::*HIS1*	This study

### Growth conditions.

Hyphal growth was induced by growing yeast cells at 30°C overnight to saturation in YEPD (2% glucose, 2% Bacto peptone [Difco], and 1% Bacto yeast extract [Difco] plus 80 mg uridine liter^−1^). The stationary-phase culture was washed with distilled water, inoculated at a 1:20 dilution into synthetic defined (SD) medium (consisting of 0.67% yeast nitrogen base [Difco], 2% [wt/vol] glucose, 80 mg of uridine liter^−1^, and 40 mg arginine and histidine liter^−1^ plus 10% calf serum [Sigma-Aldrich]), and incubated at 37°C. For live-cell imaging experiments, cells were grown on agar pads incorporating SD medium as described previously (39).

### Microscopy and image processing.

Images of exocyst components fused to YFP, and of Rom2-GFP and GFP-Rho1, were made using a DeltaVision Spectris 4.0 RT microscope (Applied Precision Instruments, Seattle, WA) with an Olympus 100× UPlanApo lens (numerical aperture [NA], 1.35; Olympus, Tokyo, Japan). Images were acquired with Softworx software, which was also used to deconvolve z-stacks where specified.

Time lapse videos of exocyst components fused to YFP and of GFP-Rho1 were captured in a single z-plane over 10 min, during which images were taken every 30 s. Abp1-YFP fluorescence was captured in a z-stack of 2 μm in which sections were 0.2 μm deep, and the z-stack was then deconvolved. All images were taken with an exposure time of 0.3 s except for images of Rom2-GFP, where a single 10-s exposure was used.

Further image processing was performed with a combination of NIH ImageJ (40) and in-house Python programs (utilizing the Python Imaging Library [PIL] module). To bring cells into the standard vertical alignment, the position of the apex of the tip and the angle of the local hyphal axis at the tip were determined visually using ImageJ. For the time series, the positions of the apex in the first and last frames were used to determine the degree of translation required per image, assuming a constant rate of growth. Images were extracted from proprietary formats using ImageJ. Python programs were then used to extract, rotate, and translate a region (typically ∼3 by 3 μm around the apex) of each image and also to generate an average image via a simple pixel-by-pixel summation process.

To create final images, and to lay out multiple images, Python programs were used in conjunction with an in-house PostScript-based plotting system, jplot. Selected threshold levels were linearly mapped onto the full RGB (red-green-blue) intensity range in the output images. For the overlays, an opaque overlay method was used to avoid distortion. All other graphs were also made with jplot. ImageMagick (www.ImageMagick.org) or Ghostscript was used to convert PostScript images to bitmaps.

### Image quantitation.

To measure the variability of the protein distribution in the time series, an automated tracing method was implemented. A point along the hyphal axis approximately one hyphal width back from the apex was chosen as an origin. A line was then traced out from the origin at an angle θ to the axis, and the point of maximum fluorescence intensity was found. The angle θ was then swept from −120° to 120° to produce the graphs in Fig. S1 in the supplemental material.

In order to allow a direct comparison between measurements made in different experiments where small variations in hyphal width occur (and to facilitate comparison with other organisms), we normalize all measurement of σ to the width of the hypha in which the measurement was made, and we express the results in hyphal width units (hwu). To obtain protein distributions as a function of arc length from the tip, for use in calculations, a trace was made around the average images in ImageJ. The coordinates of the trace were exported, and Python programs were used for extracting the intensity profile. The profiles were then fitted to a Gaussian function, allowing the intensity, width, and baseline offset of the function to be varied. Fitting was performed using the Levenberg Marquardt algorithm implemented in an in-house fitting program using Numerical Recipes routines.

### Model calculations.

All hyphal growth calculations were performed using Python programs (the code used is presented in Table S2 in the supplemental material). For practical reasons, length units were considered to be in μm and were converted to hwu following the calculations. Final production calculations were run for 5,000 steps, initialized with 1,200 annular regions initially, with a central disc with a radius of 0.05 μm, and with equal spacing of 0.001 μm. The other parameters were σ (0.46 μm [measured from hyphae with radii of 1.7 μm; thus, σ is equal to 0.27 hwu]), γ (0.01), and ε (0.005). *s*_endo_ and ϕ were set as described below. A new annular region was shed from the central region every 5 steps, maintaining *r_i_*/*l_i_* at >50. For the illustrative examples in Fig. 4, a much coarser division into regions was used. These calculations were run with 30 annular regions initially, with a central disc with a radius of 0.2 μm, initial annular spacing of 0.1 μm, a σ of 0.4 μm, a γ of 0.01, and an ε of 0.005. For Fig. 4B, the value of *s*_endo_ was 0.85 μm and the ϕ value was 0.005. A new annular region was shed from the central region every 200 steps.

In the model, ε is a scaling constant that subsumes various factors such as the number of synthase molecules per vesicle and the relationships between the unit of *A_i_* and the arbitrary units of *E_i_* and *S_i_*. γ is a scaling constant that subsumes factors such as the rate of synthesis per synthase molecule, the cell wall thickness, and conversion factors involving the units of various quantities. In the calculations, the combined factor $\sqrt{\varepsilon \gamma }$ simply scales the amount of time represented by a time step and does not affect the form of the hypha. For example, the identical form is created if ε is doubled and γ is halved. Thus, it is important that the factor εγ is small enough that the amount of growth per time step is small. In our calculations, we set the time step δt to 1 (in arbitrary units). Both *E_i_* and *S_i_* are best thought of as proportional to the numbers of molecules per unit area, although it will never be necessary (or possible) to specify their absolute units in this work, since such units could equally be subsumed into the parameters ε and γ. Because ϕ is defined as a value per time step, its interpretation is linked to the issue of the definition of the meaning of the time step, and hence to the particular choice of ε and γ. Therefore, it is best interpreted in terms of the range of arc length over which a significant drop in synthase density occurs due to endocytosis.

### Raytracing.

Raytraced images were made using POV-Ray (www.povray.org). The output files from calculation time points were converted to POV-Ray primitive objects (cone and torus) using Python programs. The coloring of the primitive objects was based on the appropriate protein intensity (normalized to 1 at its maximum) linearly mapped onto 0 to 240° of the hue range of the HSL (hue, saturation, and lightness) representation of RGB color space.

## RESULTS

### Determination of protein distributions.

In order to parameterize and test our model, it was necessary to measure the distributions of a number of fluorescently tagged proteins in the tip.

### Exocyst components.

**Fig 1:**
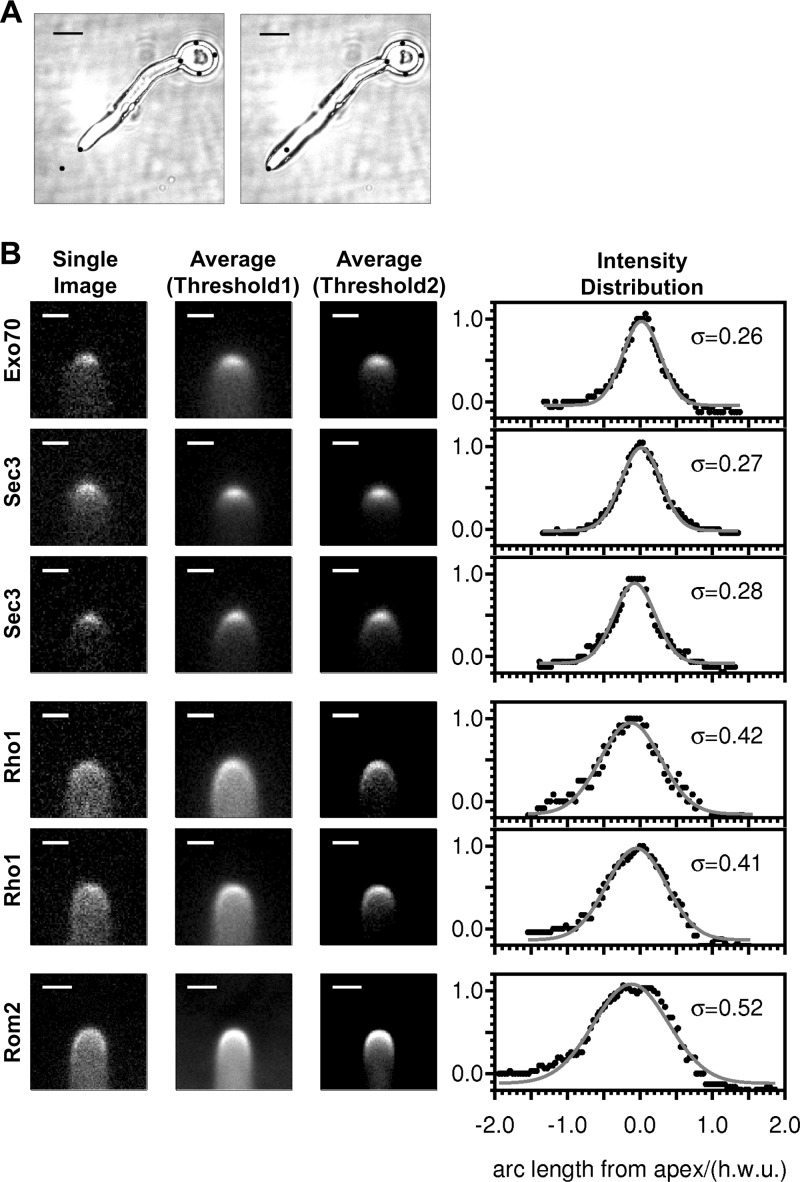
Quantitation of protein distributions. (A) Differential interference contrast images showing the growth of a C. albicans hypha from time zero (left) to 10 min (right). The marker spots are at the same positions in the two images and show that the mother cell body does not move significantly in this time, while the tip moves through approximately 2 hyphal diameters. Bar, 5 μm. (B) Fluorescence images and quantitation of fluorescence intensity in live cells. Data are shown from time series for three cells expressing tagged exocyst components (Exo70, Sec3) and two cells expressing tagged Rho1 and from a collection of cells expressing Rom2-GFP. Leftmost panels show representative single images either from the time series (Exo70, Sec3, Rho1) or from a single cell (Rom2), with the minimum intensity set to the background outside the cell (plus 1 standard deviation). Central and right panels show average images from the time series (Exo70, Sec3, Rho1) or from a collection of 42 cell images (Rom2) with two threshold settings. In the central panels, the minimum intensity is set at the background outside the cell (plus 1 standard deviation), whereas in the panels on the right, the minimum intensity is set at the background cytoplasmic level away from the apex. Two thresholds are shown because the cytoplasmic background is much higher in the Rho1 and Rom2 images, obscuring the crescent at the tip. Bars, 1 μm. The graph on the right shows the fluorescence intensity traced around the hyphal tip (black dots) and the fit of these data to Gaussian curves (solid curves), plotted against the arc length from the tip of the cell. The value of σ for each fitted curve is shown. The fluorescence data were normalized to 1 at the maximum and to zero far from the apex. The units for arc length and σ are hyphal width units (hwu), in which the width of the hypha is 1 unit (see the text).

In our first observations of GFP-tagged exocyst components made on rather short time scales (<0.2 s), we frequently observed that the distribution of exocyst density was not symmetrically arranged around the growth axis and that there was also a variation in the width of the distribution in different images. In order to test whether the asymmetry and variation in exocyst distribution were static and variable between cells, or were dynamic and more uniform between cells when time-averaged, we recorded time lapse videos, collecting frames every 30 s over a period of 10 min. We then analyzed data for cells that grew straight during this period; a representative cell is shown in Fig. 1A. For each such data set, we applied a rotation to the images to bring the hypha into a standard vertical orientation. We also used the initial and final tip positions to apply a translation (linearly scaled with time) to each image. This simple method was found sufficient to bring all images from a series into alignment during a time in which each hypha grows by approximately 2 hyphal diameters. The full data sets (see Fig. S1 in the supplemental material) confirm that the position of the maximum exocyst density is variable in time, a behavior we have observed repeatedly in all data sets collected from cells expressing Sec3-YFP, Sec8-YFP, Exo70-YFP, and Exo84-YFP.

To provide a measure of exocyst density over a time period in which the hypha grows significantly, the images within each series were therefore averaged. Resulting average images are shown in Fig. 1B for Exo70-YFP and for two separate experiments using Sec3-YFP. The distribution of intensity was traced around each average image and was found to fit adequately to a Gaussian function. In order to allow a direct comparison between measurements made in different experiments where small variations in hyphal width occur (and to facilitate comparison with other organisms), we normalized all measurements of σ to the width of the hypha in which the measurement was made. In such hyphal width units (hwu), the fits for the three images for exocyst components shown in Fig. 1B give σ in a range of 0.26 to 0.28 hwu. As an example, a value for σ of 0.27 hwu corresponds to a σ of 0.46 μm in a hypha with a width of 1.7 μm.

### GS and Rho1.

To measure the distribution of GS activity, we first attempted to determine the distribution of the localization of Gsl21-GFP. Gsl21 is one of three proteins encoded in the C. albicans genome homologous to S. cerevisiae genes encoding GS. However, we found that that its localization was anomalous, since it appeared be trapped in an intracellular membranous compartment.

GS is activated by a GTPase, Rho1 (41). In secretory vesicles, GS is complexed with Rho1-GDP, and Rho1 is converted to the active GTP-bound form by its GEF Rom2 only after fusion with the plasma membrane (42). In turn, Rom2 interacts with the cell wall stress sensors Mid2 and Wsc1 to Wsc3 (43). Rom2 is one of two GEFs for Rho1, along with Tus1. However, Rom2 has been shown to be the specialized GEF for Rho1 in its GS-activating role (44). We therefore used GFP-Rho1 as a surrogate marker of GS localization. The data for GFP-Rho1 were collected in the same fashion as those for the exocyst components. The distributions for GFP-Rho1 are considerably broader than those for the exocyst components, with a σ of ∼0.4 hwu.

### Rom2.

We were unable to delete the second copy of Rho1 in the *RHO1/GFP-RHO1* strain, raising doubts as to whether GFP-Rho1 is fully functional. Moreover, GS is active only when Rho1 is in its GTP-bound form, for which GFP-Rho1 is not informative. Because of these limitations of GFP-Rho1, we also measured the fluorescence of a functional Rom2-GFP strain. The fluorescence signal of Rom2-GFP was faint; nevertheless, upon long exposure, a broad crescent was evident (Fig. 1B). These long exposures bleached the GFP signal, precluding the repeated exposures necessary for time lapse movies, which we used to quantitate the fluorescence for the exocyst proteins. The data for Rom2-GFP were therefore obtained by averaging the images of a number of individual cells (*n* = 42). The distribution is even broader than that for GFP-Rho1, with a σ of ∼0.5 hwu.

### Growth model.

**Fig 2:**
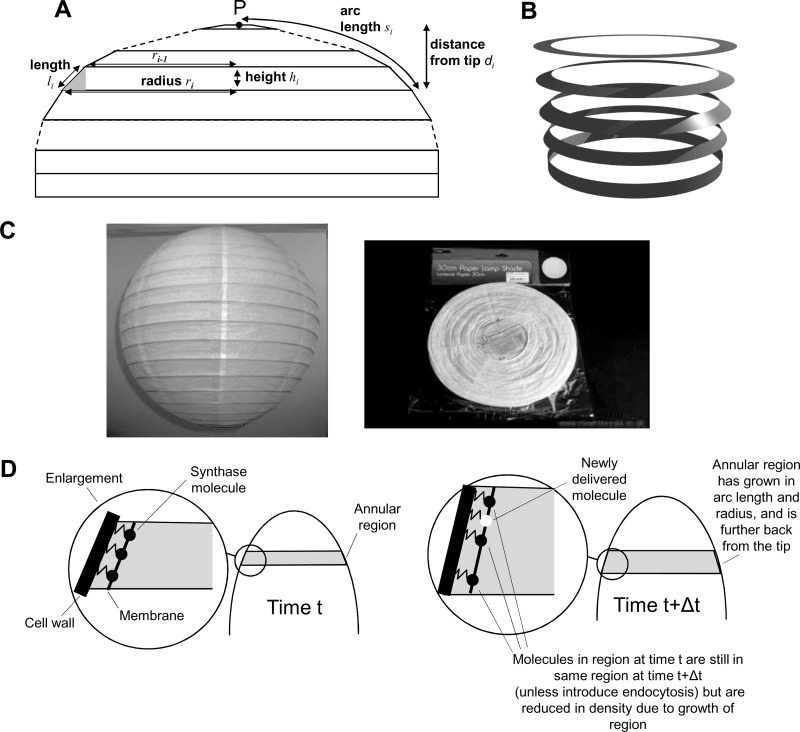
Hyphal geometry. (A) Geometry. We treat the hypha as a set of concentric annular regions. Provided the hypha remains inflated by turgor pressure and the subdivision into annuli is sufficiently fine, each annular region approximates well to the form of a truncated conical shell with open circular ends, which is most conveniently characterized by two variables, the radius, *r_i_*, and the arc length, *l_i_*. Conversely, if the values of *r_i_* and *l_i_* are specified for all of the annular regions, the entire shape of the hypha can be calculated. The height of the truncated cone, and hence the contribution to the extension of the hypha, is readily calculated by applying Pythagoras' theorem to the shaded triangle: $h_{i}=\sqrt{l_{i}^{2}-{\left(r_{i}-r_{i-1}\right)}^{2}}$. The distance of each annular region *i* from the tip can then be calculated as $d_{i}=\underset{j\leq i}{\sum }h_{j}$, the arc length round to an annular region is calculated as $s_{i}=\underset{j\leq i}{\sum }l_{j}$, and the surface area of one face of the shell, *A_i_*, is given by 2π*l_i_r_i_* (where *l_i_* is ≪*r_i_*). The very apex of the tip is described by a small circular region. (B) A narrow annular region can be freely rotated. An annular region where *l_i_* is ≪*r_i_* can be freely deformed as shown. Where *l_i_* is ≪*r_i_*, the fractional changes in area or in the inner and outer radii of the annulus are negligible during such a deformation. (C) Analogy with a Chinese paper lampshade, shown extended (left) and in a flattened, packaged form (right). (D) Schematic representation of two time points during a calculation. A hyphal tip is shown with one annular region highlighted at time *t* (left) and at an arbitrary time Δ*t* later (right). Synthase molecules deposited up to and including time *t* are represented by black circles. A synthase molecule deposited during the time Δ*t* is depicted by a white circle. Assumption ii asserts that all molecules found in the annular region at time *t* are also found in the annular region at time *t* + Δ*t* (unless endocytosis is active). The zigzag lines represent the extrusion of a polymer into the cell wall. Since this polymer is envisaged as enmeshed with the existing polymer, the synthase molecules are unable to diffuse relative to the local cell wall.

Our treatment of hyphal geometry is shown in Fig. 2, and the set of associated variables is listed in Table 2. This treatment comes largely from the strong analogy between an inflated hypha and a Chinese paper lantern or lampshade (Fig. 2C). Such a lantern, when supplied flat (Fig. 2C, right), is wrinkled because the arc lengths (*l_i_*) of the annular regions between the hoops are larger than the differences in the radii (*r_i_*) of successive hoops (Fig. 2A). The 3-dimensional shape that the lantern takes when pulled out makes each panel taut and thus extended to the maximum allowed by *l_i_*. The wire or wood hoops in the lantern would not be necessary if its ends were sealed and it were inflated with gas.

**Table 2:** Summary of variables used in the model

Variable	Description
Fundamental geometrical entities	
*r_i_*	Radius of annular region *i*
*l_i_*	Arc length of annular region *i*
*P*	Point on which the annuli are centered
Derived geometrical variables	
*h_i_*	Height contributed by annular region *i*
*d_i_*	Distance of annular region *i* from tip
*s_i_*	Arc length from *P* to annular region *i*
*A_i_*	Surface area of one face of annular region *i*
Protein densities	
*E_i_*	Exocyst density in annular region *i*
*S_i_*	Synthase density in annular region *i*
Parameters	
σ	Standard deviation of Gaussian distribution for *E*
ε	Parameter linking exocyst density to rate of synthase exocytosis
γ	Parameter linking synthase density to rate of surface area growth
*s*_endo_	Arc length from tip at which endocytosis starts (model using equation 3b)
ϕ	Fraction of synthase that undergoes endocytosis during one time step (model using equation 3b)
δ*t*	Time step for updating the variables; acts simply to scale ε, γ, and ϕ and is therefore implicitly set to 1 in calculations

**Fig 3:**
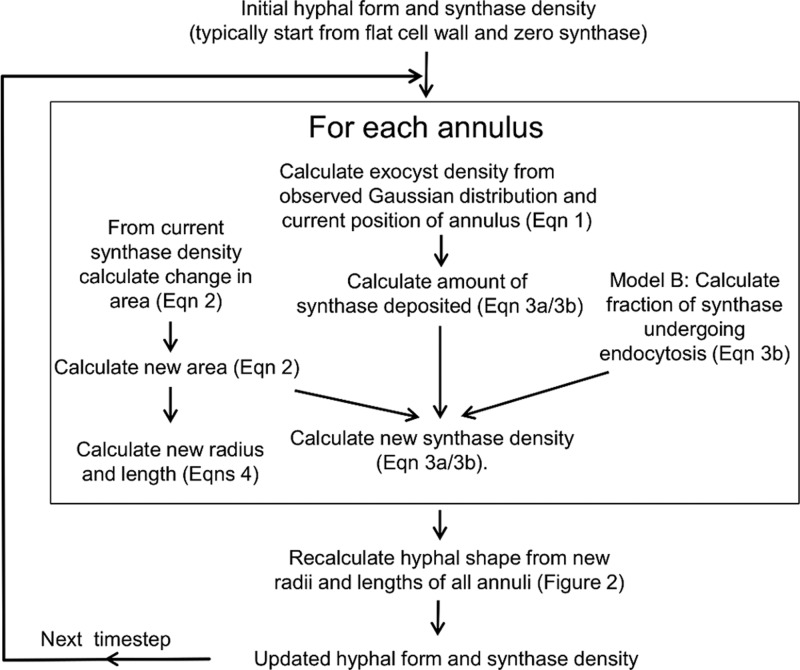
Flow chart showing the steps for updating the variables describing hyphal geometry. Given a starting set of variables, the calculations in the box are first carried out for each annulus. From the new set of *r_i_* and *l_i_* the new overall form of the hypha can be calculated (Fig. 2A). This process makes use of the fact that for an annular region where *l_i_* is ≪*r_i_*, there is freedom to alter the degree of slope of the wall so that the radius matches up with that of the preceding ring (Fig. 2B). The process can then be iterated for many time steps.

The disposition of paper in a lantern is fixed. A hypha is a much more complex structure—it is as if a lantern were having paper continually added to its panels. Therefore, in addition to these geometrical variables, each annular region is described in our model by two further variables, the local exocyst density (*E_i_*) and the synthase density (*S_i_*). The computational process for updating the variables in the model, and hence “growing” a hypha, is defined in Fig. 3, and the corresponding equations used to update the variables are given in Table 3. [Note: we use the term synthase here in a generic way to represent all enzymes that contribute to the synthesis of new cell wall material. The major component of the cell wall that determines its shape is (1,3)-β-glucan synthase, whose distribution we address experimentally. In this paper we use the term synthase in theoretical modeling of the cell wall, and we use the abbreviation GS when we refer to the experimentally determined distribution of (1,3)-β-glucan synthase.]

**Table 3:**
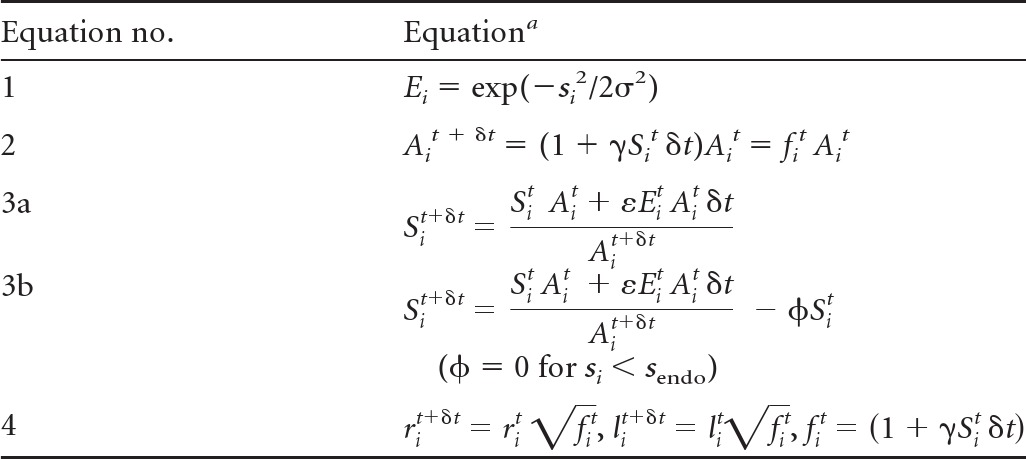
Equations of the growth model

a Throughout these equations superscript *t* or *t* + *δt* is used to indicate the value of the parameter at time *t* or *t* + *δt*. Equation 1 expresses the Gaussian distribution of exocyst density used in the model, which is parameterized from the experimental observation for σ. In equation 2, the new area of an annular region at time *t* + δ*t* is determined by calculating the amount of cell wall synthesis in the small time δ*t* (∝*S_i_^t^ A_i_^t^* δ*t*) and converting it to a change in area using the parameter γ. The two forms of equation 3 express the method by which the new density of synthase at time *t *+ δ*t* is calculated and differ only by whether endocytosis is included (equation 3b) or is neglected (equation 3a). In both forms, the amount of synthase added to an annular region in time δ*t* is ∝*E_i_^t^ A_i_^t^* δ*t* and is converted to units of *S_i_* via the parameter ε. This amount is added to the preexisting amount of synthase and is then scaled down by the new area. In this way, the decrease in synthase density due to an increase in cell wall area is included. In equation 3b, if *s* is >*s*_endo_, a fraction of synthase (ϕ) is removed per time step due to endocytosis. In equations 4, the new radii and arc lengths of annular regions are calculated assuming an isotropic mode of growth in which the radius and arc length of an annular region grow by the same factor. The parameters ε and γ do not affect the form of the hypha; they affect only the amount of growth per time step (see Materials and Methods). The very apex of the tip is represented by a small circular region. This region grows according to the same growth model as the annular regions except that it is described only by a radius and not by an arc length. After a set number of steps, the region is replaced by a combination of a new annular region and the circular region with its radius reset to its initial value.

The assumptions inherent in the equations in Table 3 are as follows. (i) The only route of entry of the cell wall-building synthase into the membrane is via the docking of vesicles, and the rate of docking of vesicles with the plasma membrane is proportional to the exocyst density (equations 3a and 3b). The exocyst density is assumed to remain constant and is approximated in the model by a Gaussian distribution (equation 1).

(ii) The synthase deposited in an annular region remains located in the same annular region (equations 3a and 3b). (Equation 3b additionally allows for the removal of synthase by endocytosis.) We propose that upon deposition in the plasma membrane, synthase molecules immediately start to synthesize cell wall polymers in the existing cell wall matrix and thereby become “locked to” the local cell wall by the enmeshing of newly synthesized cell wall material (Fig. 2D).

(iii) The synthases work at a constant activity, so that the rate of cell wall material synthesis is simply proportional to the synthase density (equation 2). This is a strong statement about the primary regulation of cell wall growth, which we consider further in Discussion.

(iv) The cell wall thickness is constant, and so the change in area is proportional to the amount of cell wall material synthesized (equation 2). This is related to assumption iii and likewise is discussed further below.

(v) The expansion of the cell wall is isotropic (equations 4). The manner in which the cell wall deforms locally upon the deposition of new material has been considered in several previous models (5, 7, 45–47). In principle, it is relatively straightforward to calculate the internal forces in the cell wall, but it is much less straightforward to calculate how the cell wall will deform, because this depends, for instance, on whether the cell wall material is considered to deform as a plastic solid or as a viscous fluid. Given the uncertainty in this area, we assume the simplest case, that the expansion is isotropic (i.e., locally the same in all directions).

(vi) We also assume that any temporary imbalance between the membrane area and the cell wall area (due to exocytosis) will be rectified by lipid flow and endocytosis and that the cell shape is dictated by the fully inflated cell wall. From assumption ii, the distribution of synthase will be unaffected by this lipid movement.

**Fig 4:**
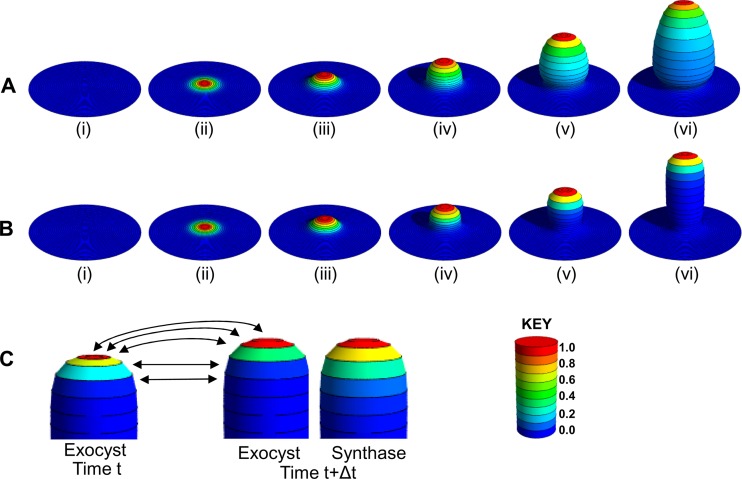
Progress of hyphal growth calculations. For the purposes of making this figure, calculations were performed with a considerably coarser subdivision of the hyphal surface than that used for regular calculations (see Materials and Methods for details). The images in this figure (and in Fig. 5C) were produced by raytracing of the hyphal forms generated by the model, as described in Materials and Methods. The hyphae are colored on a scale of zero to 1, as shown on the key cylinder. In each hypha, the maximum protein density (exocyst or synthase density according to the part of the figure) is normalized to 1. (A and B) Illustrative time courses of calculations excluding (A) or including (B) endocytosis. Hyphal forms (colored according to synthase density) before any time steps (i) and after 20 (ii), 200 (iii), 500 (iv), 1,000 (v), and 1,500 (Avi) or 2,000 (Bvi) time steps are shown. In panel A, the calculations start from a flat cell wall with no synthase in the membrane (i). After a short time (ii), the synthase density has increased due to exocytosis, and at a later time (iii), the cell wall has started to grow locally and bulge. The synthase density remains nonzero far back from the tip, and so the hypha continues to expand in diameter (iv to vi). The necking at the base is pronounced, because in the early part of the calculation, the distribution of *S_i_* is determined by the relatively narrow distribution of *E_i_*, whereas in later parts of the calculation, the distribution of *S_i_* becomes much broader. As shown in panel B, while the early parts of the calculation including endocytosis yield a hyphal form very similar to that in panel A, the distribution of synthase remains relatively narrow and settles down to a constant form (as a function of the arc length from the tip), thus giving rise to the parallel-sided hypha. A small degree of necking occurs in panel B, since the width of the distribution of *S_i_* approximately doubles from time ii to time v. (C) Illustrative positions of the hyphal tip showing the development of the size and position of annular regions and of the corresponding protein densities. The tip of the hypha is shown at time *t* and at a time Δ*t* later. The tips are colored according to exocyst density or synthase density as marked. The arrows show the correspondence between the annular regions at the two times, showing how the geometry of the regions varies in time. At the later time, the tip colored according to synthase density is shown in order to emphasize the distinction between the distributions of exocyst density and synthase density that arises in the model. The time points shown are from a calculation using the model with endocytosis.

We first consider calculations using the model incorporating equation 3a, in which no endocytosis occurs. The calculations are initialized from a flat cell wall (Fig. 4Ai) with sufficient equally spaced annular regions that the initial area of the cell wall is large compared to the cross-sectional area of the hypha that will emerge. The concentration of synthase is initialized to be zero throughout the cell wall. In the initial few time steps, the synthase concentration begins to build up in annular regions centered on point *P* in Fig. 2A, where the exocyst concentration is significant (equation 3a) (Fig. 4Aii). As the synthase levels rise, the areas of regions close to *P* increase, due to the deposition of cell wall material, and *r_i_* and *l_i_* increase according to equations 2 and 4. In contrast, at a large distance from *P*, where *E_i_* is small, synthase does not build up significantly and the annuli retain a constant radius and arc length. The only way that the enlarged radii and arc lengths of the annuli nearer *P* can be accommodated is by deformation of the planar cell wall, and in the presence of excess pressure on one side of the cell wall, the cell wall in the vicinity of *P* will bulge out (Fig. 4Aiii). The resulting height profile of the cell wall is readily calculated via the method described in Fig. 2.

**Fig 5:**
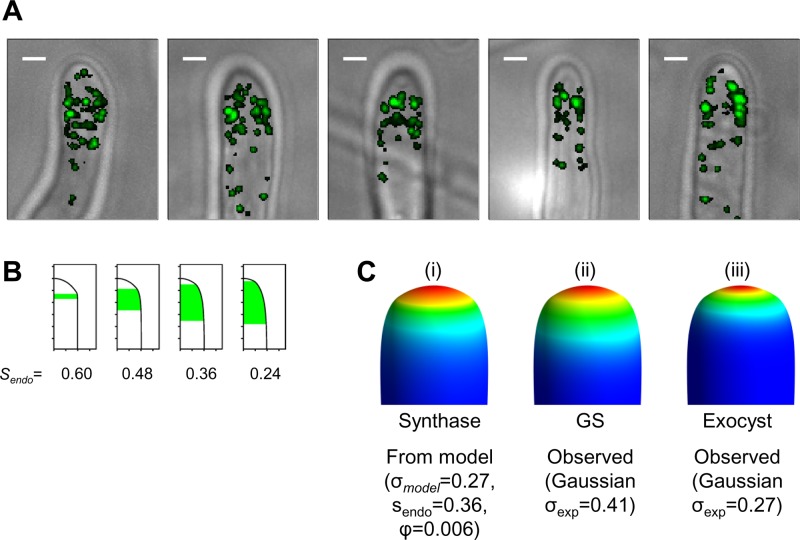
Actin patches and zones of endocytosis. (A) Fluorescence and differential interference contrast images of typical “collars” of actin patches in live C. albicans cells. Images of 5 cells are shown, with the fluorescence of Abp1-GFP overlaid onto the differential interference contrast image. For maximum contrast, the overlay is transparent for values below the minimum threshold and opaque for values above the threshold. In each image, the intense spots of fluorescence are interpreted as actin patches at sites of endocytosis events. The patches localize predominantly to a collar, although a few patches are also observed closer to the apex (e.g., in the left-hand image). Bars, 1 μm. (B) Four possible endocytosis scenarios yielding unit hyphal width. The form of the hypha (visualized as half cross-sections) is shown for calculations run with a σ of 0.27 hwu for four pairs of *s*_endo_ and ϕ values yielding a self-consistent hyphal width of 1.0 hwu. From left to right, the values for *s*_endo_ and ϕ are 0.60 hwu and 0.050, 0.48 hwu and 0.010, 0.36 hwu and 0.006, and 0.24 hwu and 0.0045, respectively. The region in green extends from the point where *s* is equal to *s*_endo_ to the point where the synthase density drops to 5% of its value when *s* is equal to *s*_endo_. Tick marks represent intervals of 0.25 hwu. (C) Raytraced 3D forms of hyphae generated with a σ of 0.27 hwu, an *s*_endo_ of 0.36 hwu, and a ϕ of 0.006. The same form is shown in each part, colored according to (i) the synthase activity (*S_i_*) predicted by the model, (ii) the observed GS activity (as inferred from Rho1 distribution and approximated to the experimental data by a Gaussian σ_exp_ of 0.41 hwu), or (iii) the observed exocyst distribution (as approximated to the experimental data by a Gaussian σ_exp_ of 0.27 hwu). The same color scheme is used as for Fig. 4.

Subsequent steps of the calculation are shown in Fig. 4Aiv to vi. Although a somewhat tubular hypha is grown, it is always thicker at the base (once an initial constriction due to the initial conditions is overcome), and it continues to grow in width without limit. This is because synthase remains in the membrane far back from the tip. It appears, therefore, that it is essential to have a mechanism for active removal of the synthase enzymes from the membrane. A role for endocytosis in the recycling of polarity proteins has been proposed previously (24, 25, 48). However, so far as we are aware, a critical role of endocytosis in removing cell wall-synthesizing enzymes has not been proposed previously. The key experimentally observed features of the actin patch distribution, as visualized by Abp1-YFP, are an absence of endocytosis close to the apex of the tip and an extensive band farther back from the tip (Fig. 5A).

We introduced a very simple model for such endocytosis by modifying equation 3a to the form in equation 3b, where *s*_endo_ is the arc length from the tip at which the band of endocytosis starts and ϕ is the fraction of synthase that is removed from the membrane by endocytosis during each time step. When ϕ is small, the zone where *S_i_* decays significantly will be broad. As ϕ is increased, the zone will become narrower. With this simple adaptation, the model generates a form of growth in which, starting from a flat membrane, the geometry close to the tip of the bulge varies in time as it distorts, but the tip rapidly takes on a form that translates without deformation as the tip moves away from the membrane (Fig. 4B). A corollary of this is that the radii of annuli as they leave the growth region become constant, and a parallel-sided tube results. Details of the way in which annular regions develop near the tip are shown in Fig. 4C.

We next examined the dependence of the predicted hyphal width on the parameters *s*_endo_ and ϕ. We used the experimentally observed value of 0.27 hwu for σ (the average of the three values determined for the distribution of the exocyst in Fig. 1) and ran a number of separate calculations with *s*_endo_ set to 0.24, 0.36, 0.48, and 0.60 hwu. For each value of *s*_endo_, we iteratively adjusted ϕ to give a self-consistent hyphal width of 1.0. The corresponding zones where *S_i_* decays significantly are shown in Fig. 5B. Assuming that such zones correspond to the zone of endocytosis as visualized by Abp1-YFP, a simple visual comparison indicates that the best agreement of the model with the distribution of actin patches is for an *s*_endo_ of ∼0.36 hwu and a ϕ of 0.006. With this choice of *s*_endo_ and ϕ, we can visualize the results by mapping the predicted distribution of synthase from the model onto the calculated 3D form of the hypha (Fig. 5Ci). This can then be compared with the observed distribution of active GS (as inferred from the distribution of Rho1) mapped onto the same 3D form (Fig. 5Cii), together with the observed distribution of the exocyst components (Fig. 5Ciii). It is clear that the model predicts a broader distribution of synthase than of the exocyst, in agreement with that observed. Comparison of Fig. 5A and B shows that the choice of an *s*_endo_ of ∼0.36 hwu is only a very approximate estimate and that a broad range of *s*_endo_ values is compatible with the observed distribution of Abp1. However, values of *s*_endo_ in the range of 0.4 to 0.8 give broadly similar degrees of agreement between the predicted synthase and observed GS distributions. As *s*_endo_ is decreased and the zone of endocytosis increases, the hyphal tip becomes more pointed (Fig. 5B). The precise forms of the hyphal tips observed experimentally are, however, quite variable and dynamic, so we do not believe that any attempt to distinguish further between these endocytosis models on the basis of current observations is warranted.

## DISCUSSION

Work endeavoring to understand the overall growth of hyphae dates back at least a century. Consequently, numerous theories have been propounded, sometimes with overlapping concepts and confusing changes of nomenclature. The reviews (and work) of Harold, Wessels, and Koch (3, 49, 50) are very useful summaries of the body of thought that predates the development of GFP-tagging methodologies. These reviews contain explanations of the soft spot, surface stress, steady-state, and vesicle supply center models. The molecular detail that has emerged from the ability to visualize the locations of specific proteins has been brought into this context by the review of Slaughter and Li (4).

In their various guises, the soft spot, surface stress, and steady-state models place as the key controlling factor in tip growth the fact that newly synthesized cell wall is more readily deformable than older cell wall. Theoretical treatments of material properties (3, 46, 47, 51) have followed from these ideas. All these models place the physical properties of the cell wall at center stage, for instance, relying on the aging (generally considered to be cross-linking over time) of the cell wall material to control the final fixing of the hyphal tube shape.

In contrast, the vesicle supply center model remains the most developed and most widely discussed quantitative model, which arguably first brought a strong cell biological focus to the control of hyphal growth. The key distinction from the surface stress model (and related models) is that while in the VSC models the deformation of the hyphal wall also arises from the effect of turgor pressure and the plasticity of the hyphal wall, this process is regulated primarily by biological control. In the VSC model, this pattern is determined by the locations of vesicle fusion events, which are calculated on the basis of the distance of the membrane from the vesicle supply center, and such events are assumed to deliver cell wall material directly.

We have presented here a model that, for the first time, incorporates the knowledge that vesicles must dock with the exocyst before fusion with the plasma membrane and that it is primarily the cell wall-synthesizing enzymes that are delivered by such events, and not the cell wall material itself. Crucially, the synthase enzymes so inserted will remain in the membrane until they are removed by endocytosis. Using the experimentally measured distribution of exocyst subunits and actin cortical patches, our model successfully predicts hyphal form. Moreover, the key prediction that the area occupied by synthase is significantly greater than that of exocyst subunits is consistent with the observed distributions of GFP-Rho1 and Rom2-GFP.

We have demonstrated that a dynamic model based on decomposition of the hypha into annular regions can be readily constructed. This methodology allows alternative biological mechanisms to be evaluated. For example, if the exocyst does not determine the location of vesicle docking, then presumably the exocyst is arriving on vesicles, and the observed distribution of the exocyst on the cell surface marks the region where vesicles are docking. Note that in such a case in the presence of diffusion, the region of docking is probably even narrower than that observed. Furthermore, an implicit or explicit assumption of many treatments of polarized growth in fungi is that the vesicles deliver new cell wall material rather the capacity to synthesize this material. A model where vesicles only deliver new cell wall material to the region marked by exocyst distribution will generate a hyphal shape, but crucially, the width of the hypha is narrower than that observed by a factor of 0.7 (see Fig. S2 in the supplemental material). To recapitulate a hypha of the width observed, the area occupied by the exocyst would have to be much more extensive than that observed and would be equivalent to the distribution of GS in Fig. 5Cii. The concrete yet mathematically simple nature of our treatment allows us to avoid descriptions that either are qualitative or are framed in the language of complex applied mathematics. For example, in Fig. 2D we use the consideration of an explicit annular region to clarify the meaning of our assumption ii, that synthase molecules remained locked to the local cell wall. The observed distributions of GFP-Rho1 and Rom2-GFP are consistent with the model incorporating this assumption; however, we cannot currently rule out the alternative possibility that synthase is able to move relative to the local cell wall. This could occur via diffusion in the membrane and/or via movement with the local membrane as it is reorganized due to the addition of new membrane following the fusion of secretory vesicles. This would require us to incorporate into the model the ability of molecules to move from one annular region to a neighboring region. This cannot be modeled without knowledge of the rates of synthase enzyme insertion at the exocyst and endocytosis at the actin cortical patches, the diffusion constant of synthase within the membrane, and the rate of lipid insertion into the membrane. At present, all of these parameters are unknown.

The maintenance of a constant cell wall thickness (assumption iv) is one of the least understood aspects of hyphal growth. It is an implicit assumption in most models of hyphal growth, including the vesicle supply model, and appears to be supported by the available experimental data. The maintenance of a constant cell wall thickness requires a delicate balance between cell wall deposition, which will thicken the wall, and cell wall deformation, which will tend to thin the wall (49). The topic is intricately bound up with the question of the regulation (or lack of regulation) of synthase and also of enzymes that can modify the physical properties of the cell wall, such as the glucanases and cross-linking enzymes.

We interpret the observation of a readily detectable presence of Rom2 with a distribution broader than that of Rho1 as evidence that all the Rho1/GS complexes are activated. Therefore, in our model, the local rate of cell wall material deposition is determined solely by the local density of synthase (assumption iii). In such a scenario, the local cell wall deformation rate must be tuned to this rate. Tuning of the local cell wall deformation rate could be achieved by appropriate control of glucanases and cross-linking enzymes.

The alternative scenario is that the local cell wall synthesis rate is tuned to the local rate of cell wall deformation. We have argued from our observations of Rom2 that our current data suggest that there is little spatial control of GS activity. This does not rule out a role for global regulation of the synthesis rate, via, for instance, regulation of the overall GS levels at a transcriptional level. In S. cerevisiae, the cell wall sensors Wsc1 and Mid2 activate the cell wall integrity (CWI) mitogen-activated protein (MAP) kinase pathway to upregulate the expression of cell wall-synthesizing enzymes after cell wall damage. In S. cerevisiae, β-1,3-glucan synthase is encoded by two genes, *FKS1* and *FKS2* (43). During unstressed growth, *FKS1* is constitutively active and is only weakly subject to CWI regulation. In contrast, *FKS2* is upregulated by the CWI pathway upon cell wall damage. This suggests that the CWI pathway may be important only under conditions of cell wall damage and not in normal cell growth (43). The interaction of Wsc1 and Mid2 with Rom2, in order to recruit Rom2 to sites of cell wall stress, may likewise be relevant only under conditions of cell wall damage. Our computational framework is very flexible and will allow us to incorporate new knowledge of these mechanisms as it becomes available.

Without a cutoff imposed by endocytosis to finally remove the synthase from the membrane, the simulations predicted hyphal swelling. We showed that a range of endocytosis parameters consistent with the observed actin patch distribution could generate hyphae of the appropriate form, although we lack sufficient information on the rates of endocytosis for specific membrane components to make the model more specific. As described above, there is considerable experimental support for a key role of endocytosis in the polarized growth of hyphae. A role for endocytosis in the recycling of the polarized growth proteins has been proposed previously (24, 25, 48). Proteins such as v-SNARES, t-SNARES, and other polarity proteins will need to be recycled, and endocytosis has been proposed to do this. However, according to our model, a key role of endocytosis is to remove cell wall-synthesizing enzymes so as to prevent continued cell wall synthesis, which would lead to hyphal swelling rather than to polarized growth. Defining this key function of endocytosis does not mean that endocytosis does not also carry out the recycling roles suggested previously. Indeed, the internalized cell wall-synthesizing enzymes could be recycled to the tip, and this could be essential for the rapid hyphal tip extension observed in many fungi, as suggested previously (48). The recycling of endocytic vesicles carrying polarity markers and cell wall-synthesizing enzymes into exocytic vesicles (52) could lead to the accumulation of vesicles that constitute the Spitzenkörper and thus explain the key role that the Spitzenkörper plays in the extremely polarized growth characteristic of fungal hyphae.

An insight provided by these simulations is that polarized outgrowth from a flat surface without endocytosis generates a structure that closely resembles the constricted neck between a mother and a bud of a yeast cell (Fig. 4A). The intuitive explanation is that in the very first stages of the outgrowth, cell wall-synthesizing enzymes are located at the site of insertion into the plasma membrane, leading to polarized growth. As this growth continues, these enzymes become located progressively farther back, leading to a more swollen structure. The initial period of polarized growth forms the narrow neck, while the later stages form the subsequent swelling at the base of the round bud. This explanation is reminiscent of an argument proposed previously (4). This raises the possibility that the variety of structures observed in the growth forms of C. albicans, such as yeast, pseudohyphal, and hyphal growth, may result from different balances between endocytosis and polarized growth. In support of this notion, we have observed that the lifetime of actin patches is shorter in hyphal cells than in yeast cells (D. Caballero-Lima and P. E. Sudbery, unpublished data), an observation that has also been reported by others (53). This may indicate that endocytosis is more active at hyphal tips than at the tips of pseudohyphal buds and buds of small yeast cells. This example illustrates how the open nature of our model allows it to be applied to situations other than the tip growth of hyphae. As discussed above, the treatment also allows one to ask clear-cut questions about which there is little knowledge at present. In particular, what is the nature of the regulation that allows cell wall synthesis and deformation to act in closely tuned harmony? If it is shown that negative regulation of synthase operates and the pattern of this regulation is experimentally determined, then equation 3b can be modified by adding a term that quantifies the regulation, in the same way in which a term was added to equation 3a to construct equation 3b, which accounts for the removal of synthase by endocytosis. Thus, as new knowledge becomes available, it can be readily incorporated into our model.

## Supplementary Material

Supplemental material
