# Supplementary material for: The Spatial Distribution of the Exocyst and Actin Cortical Patches Is Sufficient To Organize Hyphal Tip Growth
Source: Eukaryot Cell. 2013 Jul;12(7):998–1008. doi: 10.1128/EC.00085-13 (PMC3697460; doi:10.1128/EC.00085-13)
Supplement: Supplemental material [file supp_12_7_998__index.html]

Supplemental material 

# The Spatial Distribution of the Exocyst and Actin Cortical Patches Is Sufficient To Organize Hyphal Tip Growth

## 

**Files in this Data Supplement:**

- Supplemental file 1 -

  Supplemental Figures S1 and S2 and Tables S1 and S2.

  PDF, 2.3M
