# Supplementary material for: The Spatial Distribution of the Exocyst and Actin Cortical Patches Is Sufficient To Organize Hyphal Tip Growth
Source: Eukaryot Cell. 2013 Jul;12(7):998–1008. doi: 10.1128/EC.00085-13 (PMC3697460; doi:10.1128/EC.00085-13)
Supplement: Supplemental material [file EC.00085-13_zek999094122so1.pdf]

Supplementary data for Caballero-Lima et al.

Figure S1. The complete time lapse data sets for the average images shown for Exo70, Sec3 and Rho1, presented in the same order as in the main text, Figure 1B. Note how the peak of maximum intensity is not aligned to the vertical axis and how the position of the peak varies from frame to frame.

Images were recorded at 30s intervals as described in materials and methods. From left to right the columns are DIC; fluorescence of tagged protein; fluorescence of tagged protein with position of maximum fluorescence intensity along lines drawn from the filled red circle are marked with yellow filled circles; graph of fluorescence intensity (arbitrary scales) measured at the filled yellow circles, plotted as a function of angle (in degrees; vertical=0°) measured from the red filled circle. In each series frame 22 is the average of frame 1-21. In frames 1-21 the images have been translated by an amount proportional to (*image number -1*). Thus frame 1 has not been translated and frame 21 has been translated by the maximum amount. The amount of translation applied per frame was determined from the position of the cell tip in frames 1 and 21, with the assumption that the cell grows at a constant rate. The sharpness retained by the average images shows that this simple method is highly effective. Each image shows a region of 4.5x4.5 µm.

S1(a)

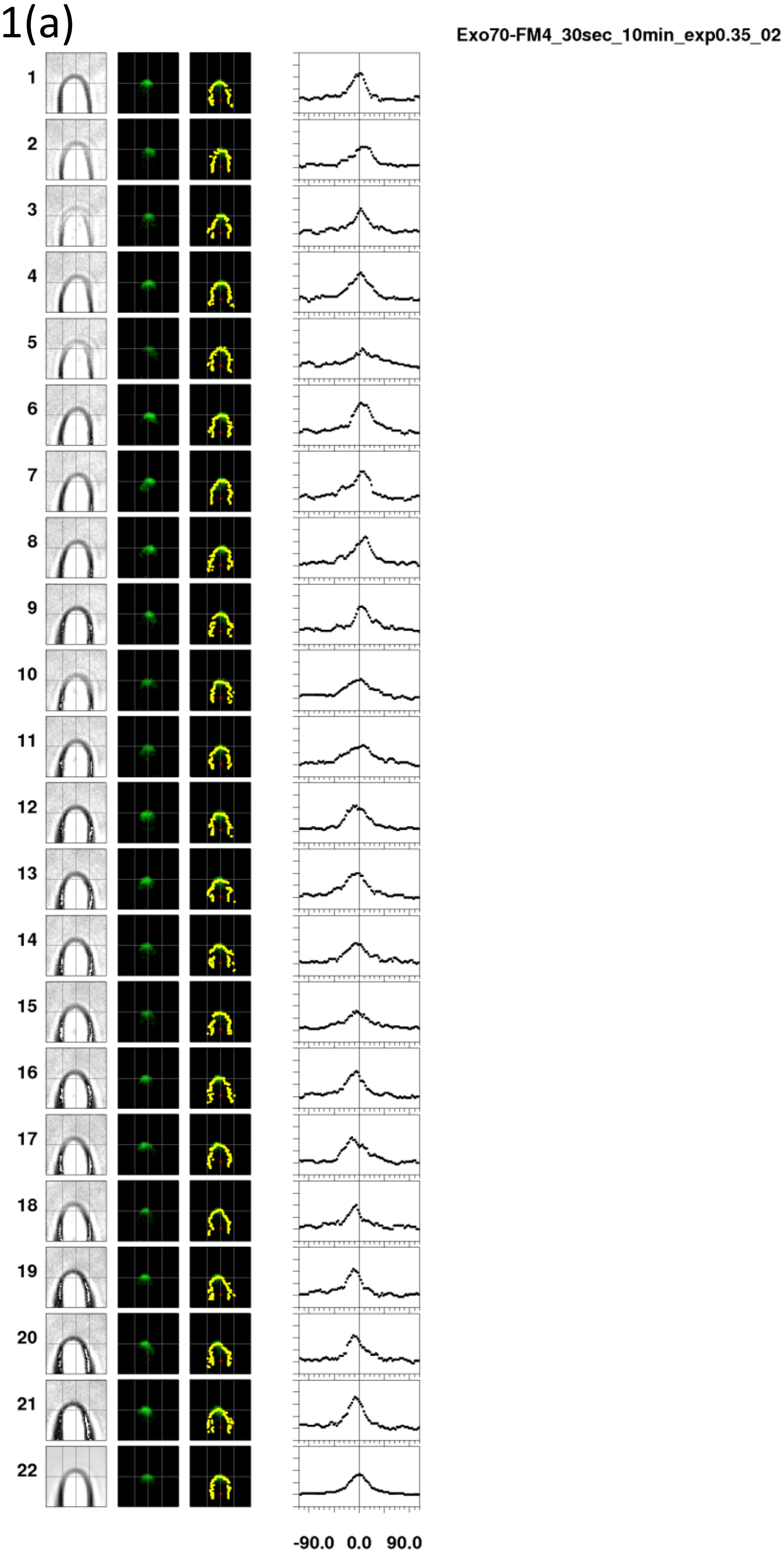
Exo70-FM4_30sec_10min_exp0.35_02
1
2
3
4
5
6
7
8
9
10
11
12
13
14
15
16
17
18
19
20
21
22
-90.0 0.0 90.0

S1(b)

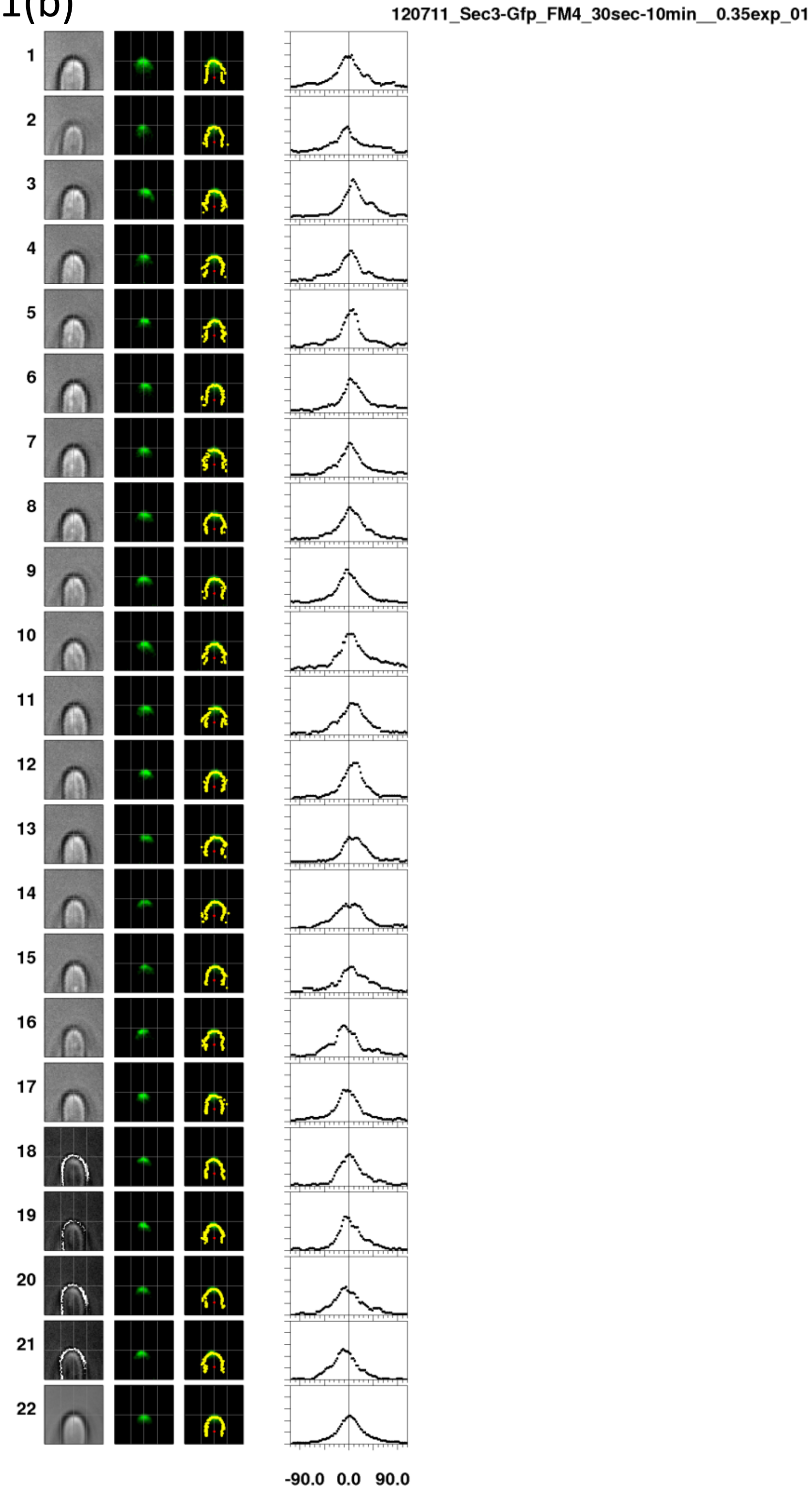
120711_Sec3-Gfp_FM4_30sec-10min__0.35exp_01
1
2
3
4
5
6
7
8
9
10
11
12
13
14
15
16
17
18
19
20
21
22
-90.0 0.0 90.0

S1(c)

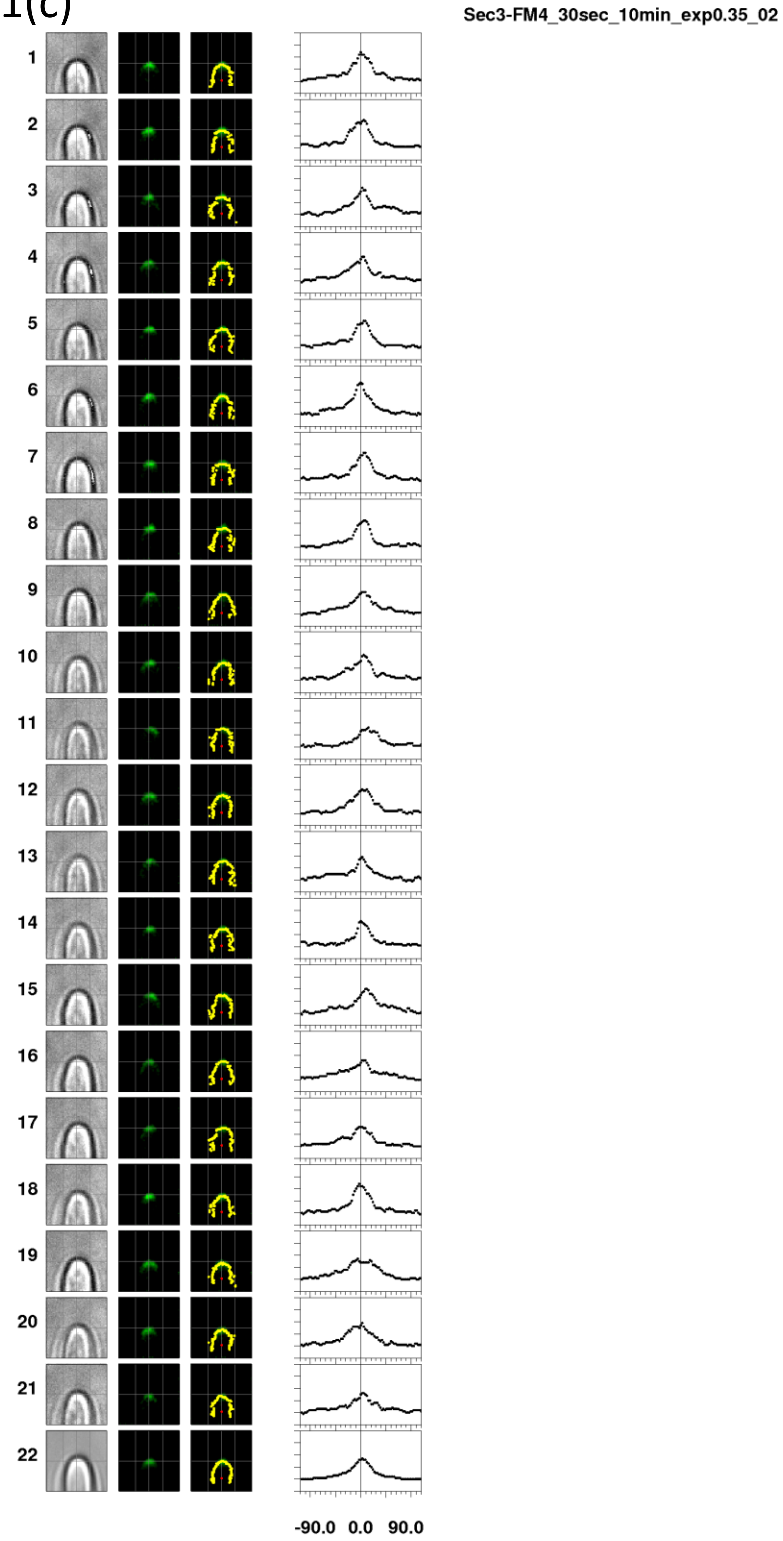
Sec3-FM4_30sec_10min_exp0.35_02
1
2
3
4
5
6
7
8
9
10
11
12
13
14
15
16
17
18
19
20
21
22
-90.0 0.0 90.0

S1(d)

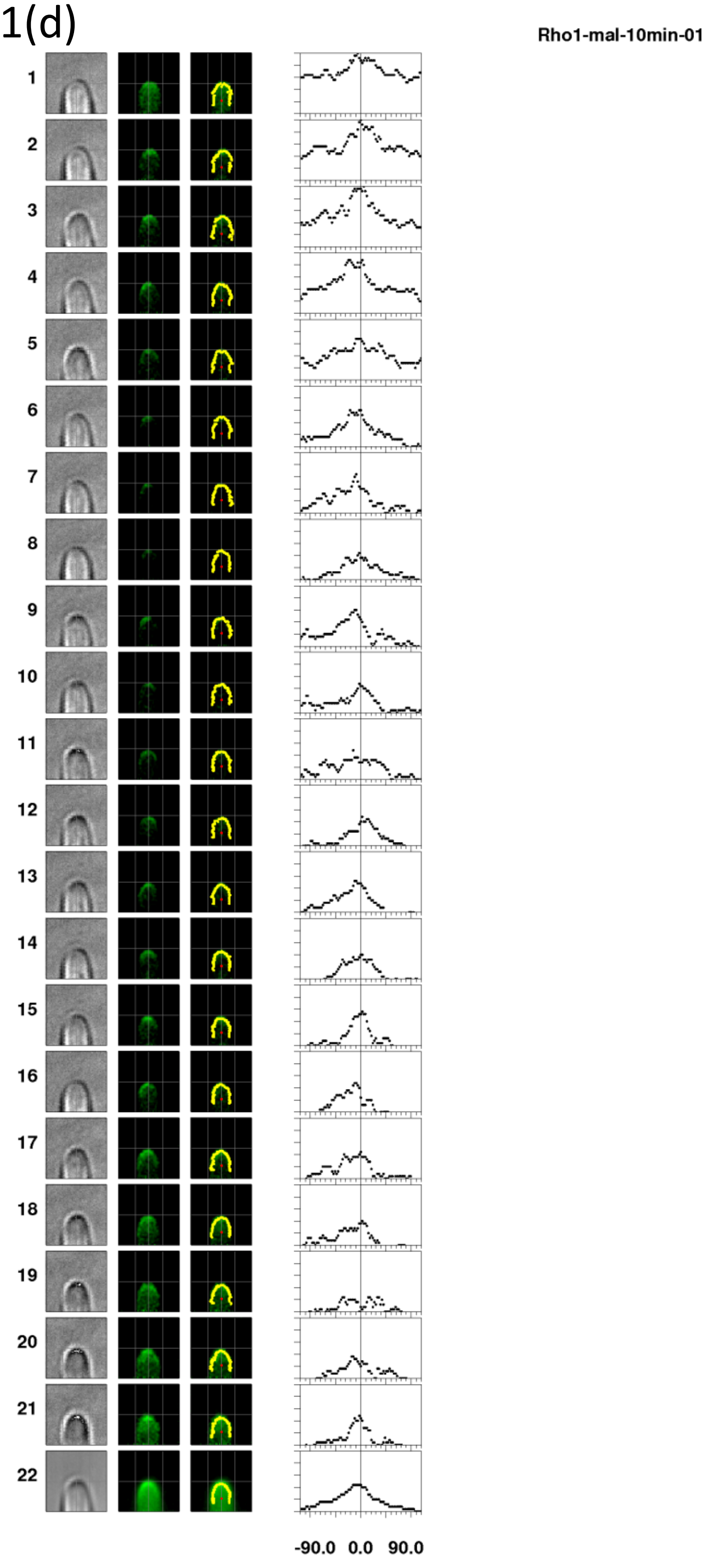

Rho1-mal-10min-01
1
2
3
4
5
6
7
8
9
10
11
12
13
14
15
16
17
18
19
20
21
22
-90.0
0.0
90.0

S1(e)

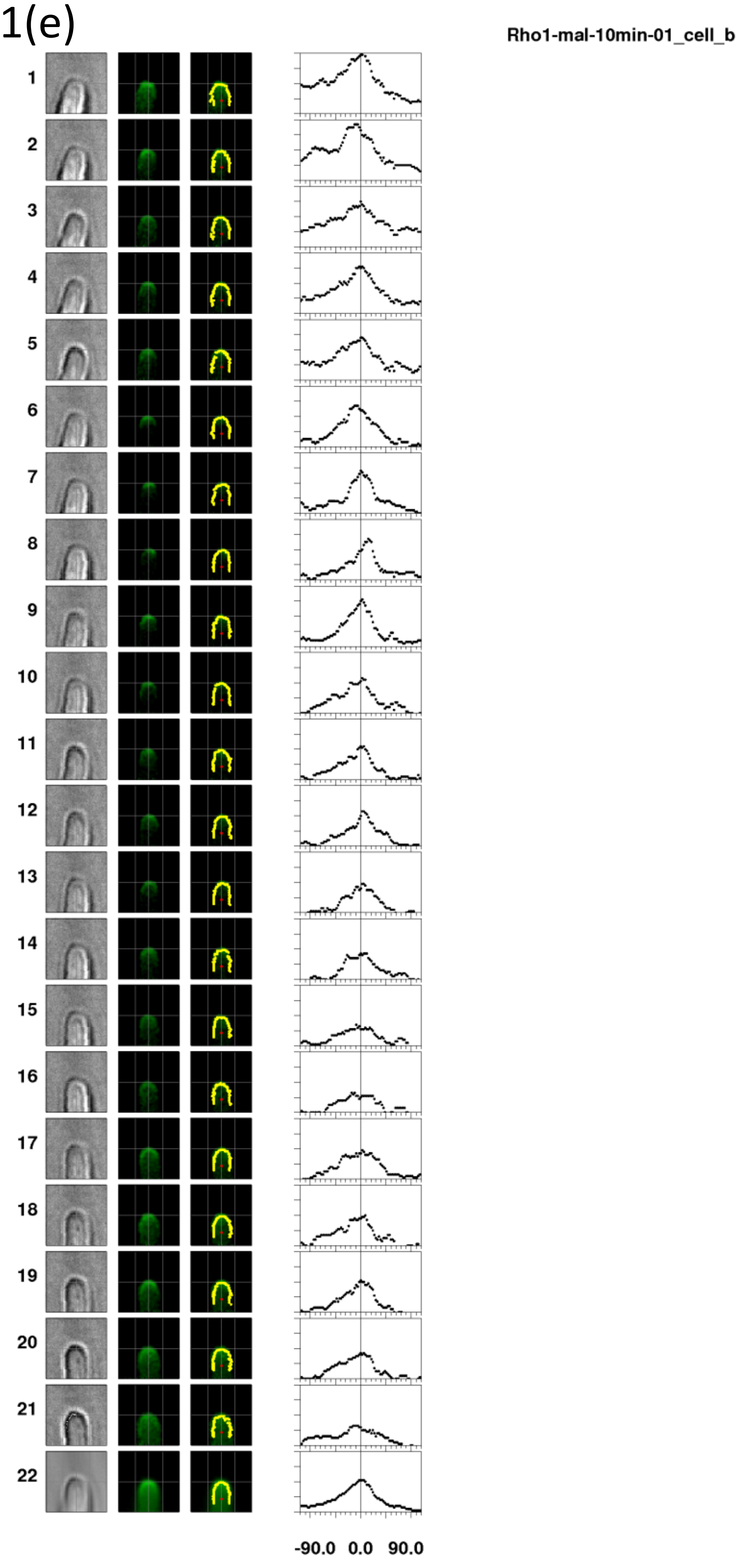
Rho1-mal-10min-01_cell_b
1
2
3
4
5
6
7
8
9
10
11
12
13
14
15
16
17
18
19
20
21
22
-90.0
0.0
90.0

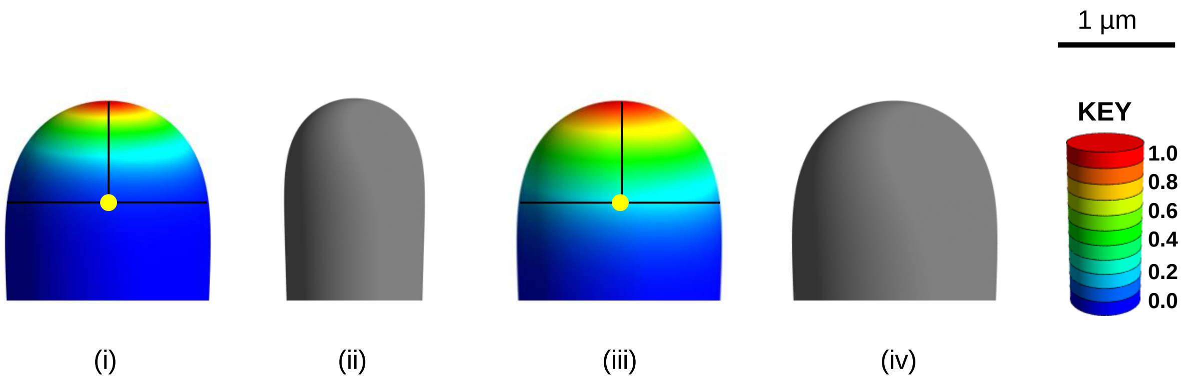

Figure S2

A model based on the exocyst delivering "surface area" directly does not generate a self consistent form, based on the experimental exocyst data. If the term in S_i in Equation 2 of Fig. 2 (main text) is replaced by E_i, then a model in which the rate of vesicle delivery directly determines the local area of surface area growth is obtained (and the calculations of S_i are irrelevant). If the experimentally observed exocyst density distribution (σ=0.45 µm, hyphal width=1.7 µm) is used in such a model (i), then the hyphal form generated by the modified model (ii) is substantially narrower (by a factor of about 0.7) than that from which the exocyst density is determined. If the value for σ used is increased to 0.68 µm a hypha of width 1.7 µm is obtained (iv). However the exocyst density implied by such a value for σ is significantly too broad (iii) compared to that observed experimentally (i).

(From very general and basic principles of dimensional analysis it can be argued that since the only parameter in this simplified model with the units of length is σ, then the width of hypha generated by a calculation must scale proportionally to σ. Thus if σ is increased by the factor 1/0.7 then a hypha of width 1.7 um is obtained).

It is also helpful to consider this in terms of the "angular" distribution of the exocyst density. In (i) and (iii) a yellow circle has been placed at a point that is at a distance from the tip equal to one radius of the hyphal tube generated. Relative to this point the exocyst density would need to subtend a much greater angle than that observed experimentally in a model in which exocyst density directly drives surface area growth.

As noted in the main text, the distribution of GS in the output from the model described in the main text (Fig. 5C(ii)) reports the pattern of the rate of local area growth, and the distribution of GS in Fig. 5 C(ii) is very similar (in angular terms) to that in (iv) above.

Table S1. Primers used in this study

Lower case shows sequence that anneals to template, upper case shows gene-specific sequence

**PRIMER**	**Nucleotide sequence (5′ → 3′)**
S1 ROM2 GFP	TGTGAAGAAAATGGATATGATATTATTATTTCCATTGATTTCTTGAATTTGAAACCAAAGTCTCCAACAggtgctggcgcaggtgcttc
S2 ROM2 GFP	AATTCCATGATAAGTTGTGTACATTAAAAACTTATACAGATTTCTTTATCTTAAAAAATACATTAGACGtctgatatcatcgatgaattcgag
S1 *MALp GFP RHO1*	CCAGGGTTAGTTGGTTGGCTGGAGAAATTGACATTACAATACTTGTTTTCTTTCTTCTTTTCGTTGTCAATTGACTTCTTTGATGGgaagcttcgtacgctgcaggtc
S2 *MALp GFP RHO1*	CAAGTCTTACCACAAGCACCATCACCGACAATGACTAATTTTCTACGAAGTTCAGCTGGACCGTTAACagcacctgcgccagcccctgcgc

This file contains the text of the program to implement the growth model, and a second helper program that can be used to extract data for a single time point in the rather lengthy output from the first program.

Both programs are intended to be run on a Unix system (Linux or a Mac) using Python2.x
With small modifications to handle input and output they would also run on Windows.

Data extracted from the second program can be plotted by your favourite means.

```
===========================================
===  Hyphal growth program (ring_growth.py)
===========================================
#!/usr/bin/python

# If this program is in file ring_growth.py
# then example usage is:
# ring_growth.py 40 2000 0.01 0.45 0.025 0.025 0.005 0 10 0.4 0.0045 20 > output_file
# the output file will contain data from the calculation, in the form of a set of python commands
# which can be read in and "exec"ed by another python program to extract information
# for a particular timestep (example provided).

import re, sys, math

nrings=int(sys.argv[1])   # number of rings used initially
nsteps=int(sys.argv[2])   # number of timesteps to run
gconst=float(sys.argv[3]) # parameter gamma
sigma=float(sys.argv[4])  # sigma for exocyst distribution
disc_radius=float(sys.argv[5]) # radius for central disc
dradius_initial=float(sys.argv[6]) # spacing of rings
enz_fac=float(sys.argv[7]) # parameter epsilon
unused_arg=int(sys.argv[8]) # unused
dstep_output=int(sys.argv[9]) # number of steps between outputs
enz_s_cutoff_inner=float(sys.argv[10]) # parameter sendo
frac_lost_per_step=float(sys.argv[11]) # parameter phi
steps_per_shedding_of_ring=float(sys.argv[12]) # number of steps between shedding
                                               # of new ring from centre

r=[]
for i in range(0,nrings):
  r.append(disc_radius+dradius_initial*i)
l=[dradius_initial]*len(r)
fac=[1.0]*len(r)
h=[0.0]*len(r)
enz=[0.0]*len(r)
exo_density=[0.0]*len(r)
s=[0.0]*len(r)

print "istep=",-1
print "nrings[istep]=",str(len(r))
print "r_record[istep]=",str(r)
print "h_record[istep]=",str(h)
print "l_record[istep]=",str(l)
```

```
print "enz_record[istep]=",str(enz)
print "s_record[istep]=",str(s)
print "exo_density_record[istep]=",str(exo_density)

steps_since_last_shed_ring=0

for istep in range(0,nsteps):

  steps_since_last_shed_ring+=1
  if steps_since_last_shed_ring==steps_per_shedding_of_ring:
    r=[disc_radius]+r
    print "#SHED",istep,steps_since_last_shed_ring,r[0],r[1],r[1]-r[0]
    l=[r[0]]+[(r[1]-r[0])]+l[1:]
    enz=[enz[0]]+enz
    fac=[1.0]+fac
    s=[0.0]+s
    exo_density=[0.0]+exo_density
    steps_since_last_shed_ring=0

  for i in range(0,len(r)-1):
    if i==0:
      s[i]=l[i]
    else:
      s[i]=s[i-1]+l[i]

    exo_density[i]=math.exp(-(s[i]*s[i]/(2*(sigma*sigma))))

    fac[i]=1.0+gconst*enz[i]
    fac[i]=math.sqrt(fac[i])

    if i==0:
      ring_area=math.pi*r[i]*r[i]
    else:
      ring_area=2*math.pi*r[i]*l[i]

    total_enz_in_ring=ring_area*enz[i]
    total_enz_in_ring+=ring_area*enz_fac*exo_density[i]
    if s[i]>=enz_s_cutoff_inner:
      total_enz_in_ring*=(1.0-frac_lost_per_step)

    r[i]*=fac[i]
    l[i]*=fac[i]

    if i==0:
      new_ring_area=math.pi*r[i]*r[i]
    else:
      new_ring_area=2*math.pi*r[i]*l[i]

    enz[i]=total_enz_in_ring/new_ring_area

  dh=[0.0]
  for i in range(1,len(r)-1):
```

```
    dhsq=l[i]**2-(r[i-1]-r[i])**2
    if dhsq>0:
      dh_val=math.sqrt(dhsq)
    elif dhsq>-1e-8:  # negative result is likely due to rounding
      print "# CORRECTION",istep,i,dhsq
      dh_val=0
      l[i]=r[i]-r[i-1] # try to prevent cumulative error
    else:
      print "dhsq too negative"
      sys.exit(1)
    dh.append(dh_val)
  dh.append(0.0)

  h=[0]*len(r)
  for i in range(len(r)-2,-1,-1):
    h[i]=h[i+1]+dh[i+1]
  if istep % dstep_output==0:
    print "istep=",istep
    print "nrings[istep]=",str(len(r))
    print "r_record[istep]=",str(r)
    print "h_record[istep]=",str(h)
    print "l_record[istep]=",str(l)
    print "enz_record[istep]=",str(enz)
    print "s_record[istep]=",str(s)
    print "exo_density_record[istep]=",str(exo_density)


===============================================================================================
===  Example extraction program to extract variables for a particular timestep from output file
===  (extract_form.py)
===============================================================================================
#!/usr/bin/python

# If this program is in file extract_form.py
# then example usage is:
#    extract_form.py output_file 100 > form_step_100
# which will print out the variables for all annular regions at timestep 100 in the file "output_file" produced from a run of ring_g
rowth.py
#
# columns in output file will be i, r-i, h-i, l-i, lsum-i (=s-i), distance from apex-i, synthase_density-i, s-i,exocyst_density-i,

import sys

filename=sys.argv[1]
istep_required=int(sys.argv[2])

nrings={}
r_record={}
h_record={}
l_record={}
s_record={}
enz_record={}
```

```
exo_density_record={}
sigma_s_record={}
sigma_phi_record={}

for line in open(filename).readlines():
  exec(line)

l=0
htip=h_record[istep_required][0]
for i in range(0,nrings[istep_required]):
  l+=l_record[istep_required][i]
  print i, r_record[istep_required][i],h_record[istep_required][i],l_record[istep_required][i],l,h_record[istep_required][i]-htip,en
z_record[istep_required][i],s_record[istep_required][i],exo_density_record[istep_required][i]
```

Supplementary Table 2. Python computer code used in modelling
